# Fibroblast Activation Protein Inhibitor Theranostics: A Huge Opportunity with its Fair Share of Oncological Pitfalls

**DOI:** 10.1055/s-0044-1787886

**Published:** 2024-06-20

**Authors:** Akram Al-Ibraheem

**Affiliations:** 1 Department of Nuclear Medicine, King Hussein Cancer Center (KHCC), Amman, Jordan; 2 Division of Nuclear Medicine, Department of Radiology and Nuclear Medicine, University of Jordan, Amman, Jordan; 3 Arab Society of Nuclear Medicine (ARSNM), Amman, Jordan

Positron emission tomography (PET) imaging, utilizing fluorodeoxyglucose (FDG) as a primary radiotracer, has significantly advanced the field of nuclear oncology. However, its effectiveness is hampered by limitations such as limited specificity and insufficient sensitivity for certain types of tumors. 1 This challenge has propelled the search for novel molecular probes to enhance or complement FDG in cancer management, particularly amidst the advancements in precision oncology and the rising rates of cancer occurrence and mortality. Recent statistics from the Global Cancer Observatory in 2020 reported an alarming figure of approximately 19.3 million new cancer cases and 10 million cancer-related deaths worldwide. 2 These findings underscore the urgent need for more effective diagnostic tools in the battle against cancer.

Cancer development, progression, and metastasis induce a myriad of dynamic changes within the tumor microenvironment, which comprises the extracellular matrix and various cell types, including cancer-associated fibroblasts (CAFs), immune cells, and vascular endothelial cells. Among these, CAFs play a vital role in tumor growth and spread by facilitating immune evasion, extracellular matrix remodeling, neoangiogenesis, and drug resistance, making them a critical focus for cancer research and treatment strategies. 3 One promising target for molecular imaging is fibroblast activation protein (FAP), which is predominantly expressed in CAFs but is minimally expressed in most normal tissues. 4 The significant role of FAP in tumor-promoting activities has made it an attractive target for the development of new diagnostic probes and therapeutic agents.

In recent years, the synthesis of quinoline-based FAP inhibitor (FAPI) has made significant advancements in nuclear medicine. These inhibitors have shown potential not only for tumor imaging but also for therapeutic applications, heralding a new era of theranostic radiopharmaceuticals. By targeting FAP, FAPIs offer a promising alternative to FDG, especially in tumors where the efficacy of FDG is limited. The introduction of therapeutic radionuclides, such as ^177^ Lu, ^90^ Y, ^64^ Cu, and ^225^ Ac, linked to FAPI molecules enables direct targeting of tumors overexpressing FAP, facilitating internal radiation therapy. 5 This approach not only enhances the specificity and sensitivity of cancer detection but also opens up new avenues for targeted radionuclide therapy. By providing a more accurate assessment of the tumor microenvironment and enabling targeted therapy, FAPIs show promise for improving patient outcomes and advancing the field of cancer care. As research continues to evolve, the potential of FAPIs in the diagnosis and management of various cancer types remains a source of optimism for patients and health care providers alike, offering a more personalized and effective approach to cancer treatment.

The increasing incidence of cancer and increasing mortality rates from the top 5 major cancer types have prompted the exploration of novel molecular theranostics probes as a revolutionary concept in cancer research. 6 This has led to a significant amount of research and clinical investigation comparing FAPI imaging to commonly used FDG imaging. Studies have shown that FAPI PET imaging is particularly superior to FDG for detecting tumors with a strong desmoplastic reaction, such as breast, colon, hepatobiliary, gastrointestinal, pancreatic, sarcomatous, and rare neoplasms, making it a potential rival to FDG imaging in several lethal cancer subtypes. 7 8 9 Ongoing research will continue to explore the potential of FAPI imaging in various cancer subtypes, and clinical trials are being conducted to assess its safety and efficacy as a therapeutic tool in the coming years. 10

Despite their potential benefits, FAPI imaging faces inherent challenges and limitations. 11 Diagnostically, the FAPI has been found to have suboptimal specificity for detecting malignant tumors. The interindividual variability underscores the varying levels of FAP expression in different diseases and even within the same disease. This variability poses a challenge for the widespread use of FAP-targeting theranostic radiopharmaceuticals. Additionally, noncancerous conditions involving fibrosis can also express FAPI, which is associated with processes such as tissue repair, matrix remodeling, and fibrosis. Activated fibroblasts can be present in various conditions, including scar formation; chronic inflammatory diseases; fibrosis in organs such as the liver, kidney, and lung; and benign tumors. 8 Therefore, clinicians must consider various conditions when interpreting images obtained with FAP-targeting tracers. A recent systematic review highlighted the prevalence of nononcological FAPI uptake in the cardiovascular system, indicating vascular disorders and atherosclerosis. Additionally, the review identified instances of FAPI uptake associated with inflammation or fibrotic diseases in various organs, including the dental mucosa, thyroid gland, heart, lung, gastrointestinal tract, liver, pancreas, kidneys, bones, joints, and muscles. 12 Another recently published pictorial analysis highlighted the occurrence of FAPI-avid degenerative lesions in more than 80% of examined patients in their retrospective analysis of 91 patients who underwent whole-body PET/computed tomography using either FAPI-04 or FAPI-46 radiotracers. The most frequent degenerative lesions were found in joints and vertebral bones, which were observed in 51.6% of patients, and head-and-neck regions, which were noted in 45.1% of patients. Uterine uptake was also significant, especially in younger women, showing a strong negative correlation with age. 13 This study highlighted the importance of recognizing these pitfalls to avoid the misinterpretation of FAPI PET imaging. Therefore, it is important to be aware that numerous benign conditions may exhibit FAPI uptake, underscoring the need for caution when interpreting FAPI PET imaging results in cancer patients.

With the increasing prevalence of noncommunicable diseases and the growing interest in noninvasive assessments of autoimmune, ischemic, and degenerative conditions, some clinicians have examined the nononcological applications of FAPI and sought to explore its potential in this area. It has been observed that FAPI uptake is correlated with kidney function impairment, liver fibrosis, renal fibrosis, intestinal fibrosis, inflammatory bowel disease, and cardiac ischemia. 14 Studies have also indicated that the FAPI may have the ability to distinguish between malignant and benign conditions based on uptake patterns, and tracer uptake kinetics. 15 Recent research has highlighted the potential of the FAPI for noninvasive assessment of various systemic diseases, including immunoglobulin G4-related diseases, rheumatoid arthritis, myocarditis, and pancreatitis. 8

Currently, there is a growing focus on the multifaceted application of FAPI agents, extending beyond its traditional use in oncology. Alongside its established diagnostic capabilities, ongoing research is exploring its potential therapeutic uses, contingent upon the establishment of safety and efficacy profiles from pending clinical trials. Healthcare professionals from various nononcological disciplines are also investigating the role of FAPI PET imaging in noncancerous diseases, aiming to enhance and broaden its nononcological utilities. Thus, it is imperative for nuclear medicine physicians and theranosticians to stay updated on advancements in this evolving field, thoroughly evaluating the benefits and drawbacks to ensure optimal patient care and improve reporting practices.
